# Long-term Nonskeletal Complications in Patients With Thyroid Cancer and Hypoparathyroidism Post Total Thyroidectomy

**DOI:** 10.1210/clinem/dgaf213

**Published:** 2025-04-02

**Authors:** Eu Jeong Ku, Jooyoung Lee, Won Sang Yoo, Janghyeon Bae, Eun Kyung Lee, Hwa Young Ahn

**Affiliations:** Department of Internal Medicine, Seoul National University Hospital Healthcare System Gangnam Center, Seoul 06236, Korea; Department of Applied Statistics, Chung-Ang University, Seoul 06974, Korea; Department of Internal Medicine, Dankook University College of Medicine, Cheonan 31116, Korea; Department of Applied Statistics, Chung-Ang University, Seoul 06974, Korea; Department of Internal Medicine, Center for Thyroid Cancer, National Cancer Center, Goyang 10408, Korea; Department of Internal Medicine, Chung-Ang University College of Medicine, Seoul 06973, Korea

**Keywords:** thyroid cancer, hypoparathyroidism, cardiovascular risk, diabetes mellitus, cataract, nonskeletal complication

## Abstract

**Context:**

Thyroid cancer (TC) is a prevalent endocrine malignancy with rising incidence attributed to advancements in diagnostic technology. Despite its generally favorable prognosis, postsurgical complications, including hypoparathyroidism, can cause long-term health challenges.

**Objective:**

This study evaluated the risk of nonskeletal complications in patients with TC with hypoparathyroidism (TC with hypoP).

**Methods:**

A retrospective cohort study was conducted using the National Health Insurance Service-National Sample Cohort (2002-2019), including patients with TC diagnosed between 2006 and 2019. Participants were categorized into TC with hypoP, TC without hypoparathyroidism (TC without hypoP), and matched controls. Propensity score matching and Cox proportional hazards models evaluated the incidence and risk of nonskeletal complications, including diabetes mellitus, dyslipidemia, cardiovascular and renal outcomes, and cataracts.

**Results:**

This study included 430 and 850 patients in the TC with hypoP and TC without hypoP groups, respectively, and their matched controls. The TC with hypoP group showed significantly higher risks of diabetes mellitus (HR 1.31, 95% CI 1.01-1.68), dyslipidemia (HR 1.29, 95% CI 1.06-1.57), urinary stones (HR 1.61, 95% CI 1.00-2.57), and cataracts (HR 1.50, 95% CI 1.15-1.95) than controls (all *P* < .05). Hypertension risk was higher in the TC with hypoP group vs the TC without hypoP group (HR 1.39, 95% CI 1.00-1.93, *P* = .048). Women had higher urinary stone risk, while cataract risk increased in patients aged over 50.

**Conclusion:**

Patients with TC with hypoP are at an increased risk for specific nonskeletal complications, particularly older adults and women. These findings underscore the need for targeted monitoring and management strategies in this population. Further prospective studies are warranted to validate these associations and elucidate the underlying mechanisms.

Thyroid cancer (TC) is one of the most prevalent endocrine malignancies worldwide, with its incidence rising rapidly in recent years, largely due to advancements in diagnostic imaging techniques, particularly high-resolution ultrasound (1). Differentiated TC, which constitutes the majority of TC cases, generally has a favorable prognosis with high survival rates. However, treatment-related complications remain a major concern. Among these, hypoparathyroidism, a frequent complication following TC surgery, significantly impacts the long-term health and quality of life of patients. Postsurgical hypoparathyroidism is reported in 7% to 36% of patients, with most experiencing a temporary form that resolves within 6 months. However, approximately 1% to 5% of patients develop permanent hypoparathyroidism, which persists beyond 6 months (2).

Hypoparathyroidism results in various symptoms and complications associated with reduced blood calcium levels. Typical symptoms include paresthesia, muscle cramps, tetany, fatigue, and mental confusion caused by hypocalcemia (3). Over time, chronic hypoparathyroidism can lead to serious health issues, including compromised bone health and an increased risk of nonskeletal complications, such as urinary stones, chronic kidney disease, cardiovascular disease, and infections (4).

Previous studies have compared the complications associated with postsurgical hypoparathyroidism with those observed in healthy controls (5-7) or patients with TC without postsurgical hypoparathyroidism (8, 9). Despite extensive research, no studies have conclusively established the risk of nonskeletal outcomes among patients with TC with hypoparathyroidism (TC with hypoP) compared with that among patients with TC without postsurgical hypoparathyroidism (TC without hypoP) or matched general controls.

The objective of this study was to evaluate the risk of developing nonskeletal complications in patients with TC with hypoP. This study aimed to provide insights into the long-term health risks, informing targeted management strategies for this at-risk population.

## Materials and Methods

### Data Source

This study received an exemption from review by the Institutional Review Board (IRB) of Chung-Ang University Hospital (IRB number: 2405-013-19525), and the requirement for informed consent was waived due to the use of anonymized National Health Insurance Service (NHIS) data. The NHIS subsequently authorized access to the database after reviewing compliance with ethical and regulatory requirements (NHIS-2024-2-045). All study procedures adhered to the principles outlined in the Declaration of Helsinki. The NHIS-National Sample Cohort (NHIS-NSC) is a population-based cohort derived from the Korean NHIS, which covers nearly the entire South Korean population (10). The NHIS-NSC was established in 2006 through systematic stratified random sampling, ensuring that the cohort accurately represents the national population based on age, sex, income level, and health insurance type. For this study, we retrospectively analyzed data from 1 137 861 individuals who participated in the biennial NHIS health screening program between 2002 and 2005. The NHIS screening program provides standardized health checkups, including anthropometric measurements, blood pressure, fasting glucose, lipid profiles, and lifestyle assessments. Among this cohort, we identified 364 288 participants who were aged ≥40 years in 2006 and had undergone at least 1 health checkup (10). The NHIS-NSC methodology ensures that these individuals are representative of the general Korean population, allowing for generalizability of our findings.

### TC Group

**Figure 1.**
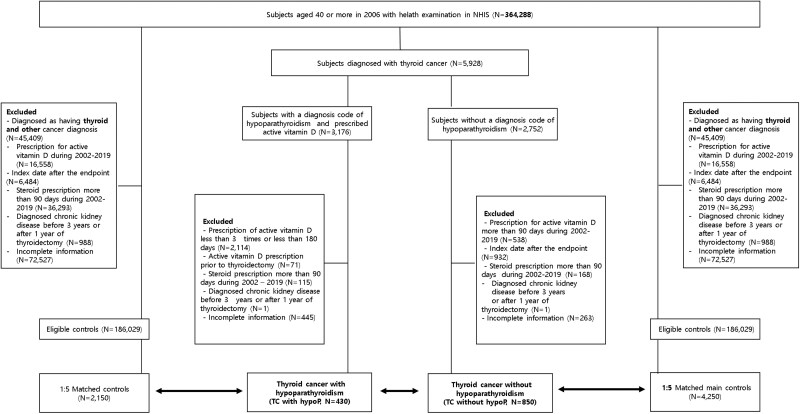
Study flow diagram for selection of study cohorts. Flow diagram depicting the selection process of study participants: thyroid cancer patients with hypoparathyroidism (TC with hypoP), thyroid cancer patients without hypoparathyroidism (TC without hypoP) following total thyroidectomy, and matched controls.

Patients with an incident TC diagnosis were identified using the International Classification of Diseases, 10th Revision (ICD-10) codes for TC (C73), documented at least twice between January 1, 2006, and December 31, 2019 (11). Among 5928 patients with TC who underwent total thyroidectomy, 3176 had a diagnosis code for hypoparathyroidism and were prescribed active vitamin D (eg, alfacalcidol, calcitriol) at least once as part of hypoparathyroidism management. Since active vitamin D is only available with a doctor's prescription in South Korea, its use is reliably recorded in the NHIS claims database. The index date for all patients in the TC group, including both TC with and without hypoparathyroidism, was set as the date of the first thyroidectomy. Exclusion criteria for transient hypoparathyroidism or other conditions affecting parathyroid function included (1) fewer than 3 active vitamin D prescriptions or prescriptions covering less than 180 days, (2) active vitamin D prescriptions before thyroidectomy, (3) steroid prescriptions exceeding 90 days between 2002 and 2019, (4) diagnosis of chronic kidney disease (CKD) 3 years before or 1 year after thyroidectomy, or (5) incomplete data (Fig. 1). Following these exclusions, 430 patients were selected to form the TC with hypoP group.

For the remaining 2752 patients with TC who did not have a diagnosis code for hypoparathyroidism, the following exclusion criteria were applied: (1) active vitamin D prescription exceeding 90 days, (2) diagnosis of TC after 2019, (3) steroid prescriptions exceeding 90 days, (4) CKD diagnosis 3 years before or 1 year after thyroidectomy, or (5) incomplete data (Fig. 1). Ultimately, 850 patients were selected to form the TC without hypoP group. The date of the first thyroidectomy was set as the index date.

### Control Group

The control group was derived from the cohort through a multistep process. First, individuals with any malignancy codes (C00-C97) from 2002 to 2019 were excluded. Second, individuals who received prescription-based active vitamin D (eg, calcitriol, alfacalcidol) during the study period were also excluded, as these medications are primarily used for hypoparathyroidism management. Third, each control group member was assigned a random index date, matching the index dates of patients in the TC group. Finally, individuals were further excluded based on the same criteria applied to the index date. This process generated 2 independent, parallel, and matched control groups from the initial control cohort for comparative analysis with the TC with hypoP and TC without hypoP groups.

### Study Outcome

We evaluated the risk of nonskeletal outcomes in patients with TC with hypoP, including diabetes mellitus, dyslipidemia, hypertension, cerebrovascular infarction, ischemic heart disease, heart failure, arrhythmia, renal stones, CKD, and cataracts. Diagnoses were established using ICD-10 codes or relevant medication prescriptions, the details of which are provided in the supplementary material (12). The study endpoint was defined as death or 10 years from the date of TC diagnosis or index date. For patients with a follow-up period of less than 10 years, the endpoint was set as December 31, 2019.

### Covariates

Demographic and health characteristics were assessed, including age at the index date, sex, income level (lower 40%, middle 30%, upper 30%), body mass index (BMI), smoking status (none, ex-smoker, current smoker), and alcohol consumption (none, mild to moderate [1-5 times a week], heavy [>6 times a week or ≥7 drinks on any day]). Clinical measures, such as systolic blood pressure (SBP), diastolic blood pressure (DBP), fasting plasma glucose (FPG), and total cholesterol level at the index date, were obtained from health examinations.

### Statistical Analysis

Baseline characteristics were presented as median (interquartile range, IQR) for continuous data and frequency (percentages) for categorical data. Three independent analyses were conducted. First, a propensity score matching analysis was performed, matching each patient in the TC with hypoP group to 5 controls using the nearest-neighbor method. The propensity score model included variables such as index date (year), age at the index date, sex, income, BMI, smoking status, alcohol consumption, SBP, DBP, FPG, and total cholesterol level. Covariate balance between groups was assessed using standardized mean differences, with standardized mean differences <10% indicating an acceptable balance (13). The crude incidence rates per 1000 person-years were calculated for each outcome. All-cause mortality was considered a competing event for nonskeletal outcomes, and cumulative incidence estimates were obtained using Aalen–Johansen estimators with the Gray test. The survival probability for all-cause mortality was estimated using Kaplan–Meier estimators with the log-rank test. Cause-specific hazard ratios (HRs) with 95% CIs were derived from cause-specific Cox proportional hazards regression models with robust SEs to account for the correlation between matched pairs. All regression models in this study were adjusted for the same set of confounders to ensure consistency across analyses, including age at the index date, sex, income, BMI, smoking status, alcohol consumption, SBP, DBP, FPG, and total cholesterol level. Second, within the TC cohort, the incidence rates and cumulative incidences of the TC with hypoP and TC without hypoP groups were compared using cause-specific Cox proportional hazards models, applying the same set of confounders. Third, each patient in the TC without hypoP group was matched to 5 controls using propensity scores, and incidence rates, cumulative incidence, and cause-specific HRs were estimated. Subgroup analyses were performed based on age group (<50 years, ≥50 years) and sex. To evaluate the reliability and consistency of our findings in relation to unmeasured confounding, E-value calculations were conducted for primary and subgroup analyses. E-values were computed for the observed hazard ratios and their CIs using the method described by VanderWeele and Ding (14).

Statistical significance was defined as *P* < .05 (2-sided). All analyses were performed using Statistical Analysis System version 9.4 (SAS Institute Inc., Cary, NC, USA) and R version 4.3.0 (R Foundation for Statistical Computing, Vienna, Austria).

## Results

### Baseline Characteristics of the Participants

Table 1 outlines the baseline characteristics of 430 patients in the TC with hypoP group, representing 7.2% of the total TC cohort (430 out of 5928). These patients were identified by a diagnosis code for hypoparathyroidism and prescribed activated vitamin D for more than 180 days. The control group comprised 2150 matched individuals (1:5 ratio). The median age of the patients in the TC with hypoP group was 55 years, and women accounted for 89.1% of this group. The median follow-up period was 5.07 years (IQR 2.19-7.38 years).

**Table 1.** Baseline characteristics of patients with thyroid cancer with postsurgical hypoparathyroidism and matched control or those without hypoparathyroidism

Baseline variable	TC with hypoP (n = 430)	1:5 Matched control (n = 2150)	Standardized difference after matching*^a^*, %	TC without hypoP (n = 850)	1:5 Matched control (n = 4250)	Standardized difference after matching*^b^*, %	*P* value*^c^*
Demographic data							
Index year, median (IQR)	2011 (2009-2013)	2011 (2009-2013)	1.1	2012 (2010-2014)	2012 (2010-2014)	0.5	.001
Age, median (IQR), years	55 (50-60)	54 (50-61)	2.5	55 (51-61)	55 (50-62)	<0.1	.154
Sex, n (%)			0.6			2.0	<.001
Women	383 (89.1)	1919 (89.3)		680 (80.0)	3343 (78.7)		
Men	47 (10.9)	231 (10.7)		170 (20.0)	907 (21.3)		
Body mass index, median (IQR)	24.2 (22.2-26.2)	24.2 (22.2-26.3)	1.4	24.0 (22.2-26.0)	23.9 (22.0-26.1)	0.8	.309
Income, n (%)			1.7			4.1	.010
Low	145 (33.7)	708 (32.9)		224 (26.4)	1196 (28.1)		
Middle	103 (24.0)	519 (24.1)		198 (23.3)	977 (23.0)		
High	182 (42.3)	923 (42.9)		428 (50.4)	2077 (48.9)		
Smoking, n (%)			1.9			1.9	<.001
None	395 (91.9)	1985 (92.3)		710 (83.5)	3533 (83.1)		
Ex-smoker	16 (3.7)	73 (3.4)		83 (9.8)	411 (9.7)		
Current	19 (4.4)	92 (4.3)		57 (6.7)	306 (7.2)		
Alcohol consumption, n (%)			2.6			2.6	.182
None	329 (76.5)	1663 (77.3)		613 (72.1)	3016 (71.0)		
Mild to moderate	74 (17.2)	364 (16.9)		183 (21.5)	957 (22.5)		
Heavy	27 (6.3)	123 (5.7)		54 (6.4)	277 (6.5)		
Clinical data							
SBP, median (IQR)	125 (113-135)	124 (112-135)	0.8	120 (111-131)	121 (111-132)	2.4	.037
DBP, median (IQR)	79 (70-82.8)	78 (70-83)	1.3	77 (70-80)	77 (70-82)	2.0	.155
FPG, median (IQR)	94 (87-105)	94 (86-103.8)	2.1	95 (87-105)	94 (87-104)	<0.1	.981
Total cholesterol, median (IQR)	195 (172-222)	196 (172-222)	1.4	194 (170-221)	195 (172-220)	0.9	.226

Abbreviations: DBP, diastolic blood pressure; FPG, fasting plasma glucose; hypoP, hypoparathyroidism; IQR, interquartile range; SBP, systolic blood pressure; TC, thyroid cancer.

^*a*^Standardized difference after matching between the TC with hypoP group and its matched control group.

^*b*^Standardized difference after matching between the TC without hypoP group and its matched control group.

^*c*^ *P* value comparing the TC with hypoP group and the TC without hypoP group.

For comparison, 850 patients were included in the TC without hypoP group (Fig. 1 and Table 1). The TC without hypoP group exhibited a lower proportion of women (80.0% vs 89.1%, *P* < .001), a higher income level (50.4% vs 42.3%, *P* = .010), and a lower proportion of nonsmokers (83.5% vs 91.9%, *P* < .001) than the TC with hypoP group. A matched control group for the TC without hypoP group was selected at a 1:5 ratio (Fig. 1). The median follow-up periods for the TC without hypoP and control groups were 4.80 years (IQR 2.23-7.28 years) and 5.17 years (IQR 2.36-7.95 years), respectively.

### Risk of Nonskeletal Outcomes Associated With Hypoparathyroidism

#### Diabetes mellitus and dyslipidemia

**Figure 2.**
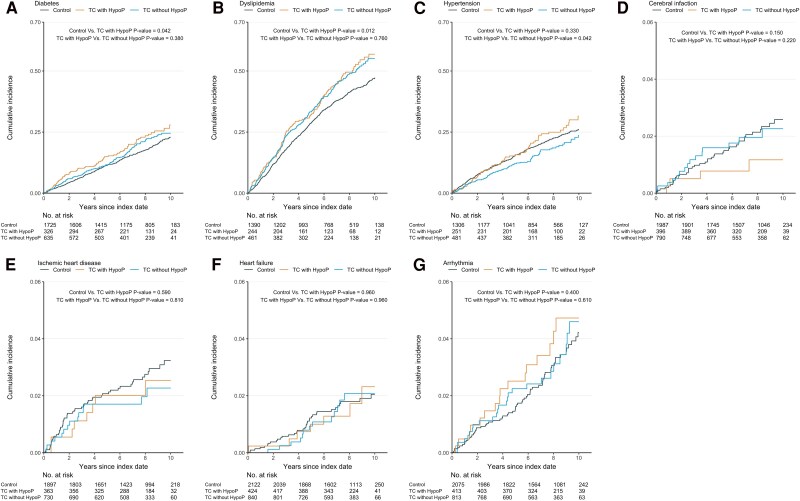
Cumulative incidence of metabolic and cardiovascular comorbidities in patients with thyroid cancer following total thyroidectomy by hypoparathyroidism Status. Cumulative incidence curves show the risk over time of developing (A) diabetes mellitus, (B) dyslipidemia, (C) hypertension, (D) cerebral infarction, (E) ischemic heart disease, (F) heart failure, and (G) arrhythmia. Groups are depicted as follows: control group, thyroid cancer patients with hypoparathyroidism (TC with hypoP), and thyroid cancer patients without hypoparathyroidism (TC without hypoP). The Kaplan–Meier method estimates cumulative incidence, with *P*-values calculated using the log-rank test. Comparisons are made between the control and TC with hypoP groups, and between the TC with hypoP and TC without hypoP groups. The number of at-risk patients at each time point is shown below each plot.

The TC with hypoP group demonstrated an increased risk of diabetes mellitus and dyslipidemia compared with the control group, with HRs of 1.31 (95% CI 1.01-1.68, *P* = .039) and 1.29 (95% CI 1.06-1.57, *P* = .011), respectively (Table 2; Table S1 (12)). In contrast, when the TC with hypoP group was compared with the TC without hypoP group, no significant difference was found in their risks for diabetes mellitus (HR 1.13, 95% CI 0.85-1.52, *P* = .404) and dyslipidemia (HR 1.02, 95% CI 0.81-1.28, *P* = .867). Furthermore, in comparison between the TC without hypoP and control groups, the risk for both diabetes mellitus and dyslipidemia remained elevated (Table 2; Table S2 (12)). The cumulative incidence curves for diabetes mellitus and dyslipidemia are shown in Fig. 2A and 2B.

**Table 2.** Risk of nonskeletal outcomes in patients with thyroid cancer with and without hypoparathyroidism compared with matched controls

Outcome variables	TC with hypoP vs matched control			TC with hypoP vs TC without hypoP			TC without hypoP vs matched control		
	HR	95% CI	*P* value	HR	95% CI	*P* value	HR	95% CI	*P* value
Metabolic									
Diabetes mellitus	1.31	1.01-1.68	.039	1.13	0.85-1.52	.404	1.27	1.04-1.56	.020
Dyslipidemia	1.29	1.06-1.57	.011	1.02	0.81-1.28	.867	1.22	1.06-1.42	.008
Cardiovascular									
Hypertension	1.14	0.87-1.50	.331	1.39	1.00-1.93	.048	0.85	0.68-1.07	.179
Stroke	0.48	0.17-1.35	.165	0.50	0.17-1.52	.223	1.66	0.67-2.02	.599
IHD	0.81	0.38-1.73	.592	1.11	0.46-2.64	.819	0.62	0.36-1.08	.090
Heart failure	0.98	0.43-2.21	.952	0.97	0.39-2.44	.951	0.96	0.52-1.75	.885
Arrhythmia	1.27	0.72-2.22	.410	1.17	0.62-2.19	.626	1.09	0.70-1.69	.718
Renal									
Urinary stone	1.61	1.00-2.57	.048	1.30	0.76-2.22	.340	1.23	0.84-1.82	.289
CKD	2.20	0.95-5.11	.066	2.43	0.84-7.02	.100	1.14	0.49-2.67	.755
Cataract	1.50	1.15-1.95	.003	1.29	0.94-1.75	.110	0.98	0.79-1.21	.820
Death	0.82	0.34-1.96	.649	1.02	0.88-1.22	.767	0.94	0.56-1.56	.807

Abbreviations: CKD, chronic kidney disease; HR, hazard ratio; hypoP, hypoparathyroidism; IHD, ischemic heart disease; TC, thyroid cancer.

#### Cardiovascular outcomes

No significant differences were observed in the risk of hypertension, stroke, ischemic heart disease, heart failure, or arrhythmia between the TC with hypoP and control groups (Table 2; Table S1 (12)). Similarly, no significant differences were detected between the TC without hypoP and control groups (Table 2; Table S2 (12)). However, the risk of hypertension was significantly higher in the TC with hypoP group than in the TC without hypoP group (HR 1.39, 95% CI 1.00-1.93, *P* = .048; Table 2; Table S3 (12)). The cumulative incidence of hypertension was also higher in the TC with hypoP group than in the TC without hypoP group (Fig. 2C).

#### Renal outcomes

**Figure 3.**
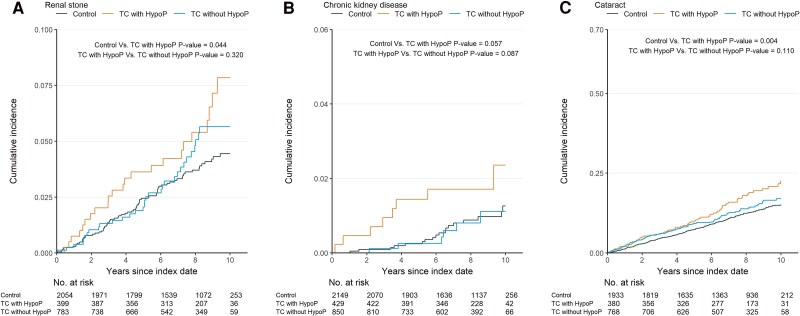
Cumulative incidence of renal stone, chronic kidney disease, and cataracts in patients with thyroid cancer following total thyroidectomy by hypoparathyroidism Status. Cumulative incidence curves display the risk over time of developing (A) renal stones, (B) chronic kidney disease, and (C) cataracts. Groups are represented as follows: control group, thyroid cancer patients with hypoparathyroidism (TC with hypoP), and thyroid cancer patients without hypoparathyroidism (TC without hypoP). The Kaplan–Meier method is used for cumulative incidence estimation, and *P* values are calculated using the log-rank test. Comparisons include the control group vs the TC with hypoP group, and the TC with hypoP group vs the TC without hypoP group. The number of at-risk patients at each time point is shown below each plot.

The TC with hypoP group demonstrated a significantly higher risk of urinary stones than the control group (HR 1.61, 95% CI 1.00-2.57, *P* = .048). Although an increased risk of CKD was observed in the TC with hypoP group vs the control group, it did not reach statistical significance. Comparisons between the TC with hypoP and TC without hypoP groups showed no significant differences in the risk of urinary stones or CKD (Table 2; Table S3 (12)). The TC without hypoP group did not exhibit an elevated risk of urinary stones or CKD compared with the control group (Fig. 3A and 3B; Table S2 (12)).

#### Cataracts

The risk of cataracts was significantly higher in the TC with hypoP group than in the control group, with an HR of 1.5 (95% CI 1.15-1.95, *P* = .003). However, no significant difference in the risk of cataracts was observed between the TC without hypoP and TC with hypoP groups (Table 2 and Fig. 3C). The TC without hypoP group did not show an increased cataract risk compared with the control group (Table 2; Table S2 (12)).

#### Subgroup analysis based on age and sex

**Figure 4.**
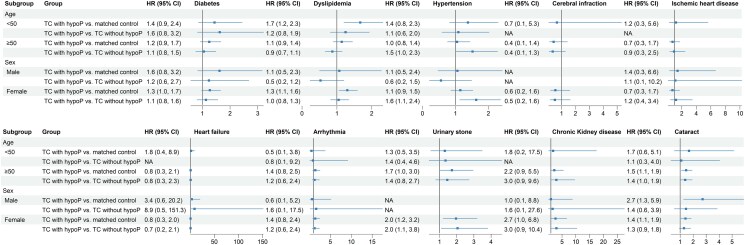
Subgroup analysis of hazard ratios (HR) for metabolic and cardiovascular disease, urinary stones, chronic kidney disease (CKD), and cataracts in patients with thyroid cancer with hypoparathyroidism (TC with hypoP). HRs with 95% confidence intervals are presented for outcomes among patients with TC with hypoP compared with matched controls and patients with thyroid cancer without hypoparathyroidism (TC without hypoP). The analysis is stratified by age (<50 and ≥50 years) and sex (male and female). An HR >1 indicates a higher risk in the specified condition for patients in the TC with hypoP group in the subgroup than in the reference group. Results of the analysis of the risk of urinary stones in men are not shown due to insufficient data in the male subgroup.

Abbreviation: NA, not available.

Among individuals aged under 50 years, there was no significant difference in the risk of metabolic, cardiovascular disease, urinary stones, CKD, and cataracts in the TC with hypoP group compared with either the control or TC without hypoP groups (Fig. 4). Among individuals aged 50 years or older, the risk of cataract in the TC with hypoP group was 1.46 times higher than that in the control group (95% CI 1.10-1.92, *P* = .008) and 1.39 times higher than that in the TC without hypoP group (95% CI 1.01-1.93, *P* = .046) (Table S4 (12)). Sex-specific analysis revealed a significantly higher risk of urinary stone for women in the TC with hypoP group (HR 1.96, 95% CI 1.21-3.19, *P* = .006 vs control; HR 2.05, 95% CI 1.10-3.81, *P* = .024 vs TC without hypoP) (Table S4 (12)). Additionally, women in the TC with hypoP group had higher risks of CKD (HR 1.96, 95% CI 1.21-3.19, *P* = .006) and cataracts (HR 2.66, 95% CI 1.05-6.75, *P* = .040) than those in the control group, although no significant differences were observed compared with those in the TC without hypoP group (Table S4 (12)).

#### Sensitivity analysis

E-value analysis was performed to assess the potential impact of unmeasured confounding. The E-values for diabetes mellitus, dyslipidemia, renal stone, and cataract in the comparison between the TC with hypoP and control groups were 1.70, 1.67, 2.60, and 2.37, respectively (Table S1). These values suggest that unmeasured confounding alone would not fully account for the observed associations unless the confounders exert exceptionally strong effects on both TC with hypoP and the respective outcomes. Similarly, in comparisons between TC with hypoP vs TC without hypoP and TC without hypoP vs matched control, the calculated E-values remained above 1.56 for all statistically significant outcomes (Tables S2 and S3 (12)).

Additional sensitivity analyses were conducted, showing that statistical significance was maintained after adjusting for baseline physical activity and evaluating the population over the complete follow-up period without a 10-year restriction (Tables S5 and S6 (12)).

## Discussion

This study evaluated the risk of nonskeletal complications in the TC with hypoP group, comparing it with matched controls and the TC without hypoP group. Our findings indicate that the TC with hypoP group had an increased risk of diabetes mellitus, dyslipidemia, urinary stones, and cataracts compared with the control group. However, hypertension was the only outcome significantly higher in the TC with hypoP group vs the TC without hypoP group.

The incidence of metabolic conditions, such as diabetes mellitus and dyslipidemia, was markedly higher in the TC with hypoP group compared with controls. A similar trend was observed in the TC without hypoP group, suggesting that the risk of these metabolic complications may be associated with TC surgery itself, regardless of parathyroid dysfunction. This is consistent with previous studies indicating an increased risk of diabetes mellitus (1.2- to 1.4-fold) and dyslipidemia (up to 1.36-fold) following TC surgery (15, 16). In TC, a potential mechanism for the increased risk of diabetes is the maintenance of a subclinical hyperthyroid state due to TSH suppression therapy. Thyroid hormones can enhance hepatic gluconeogenesis, increase lipolysis in peripheral adipose tissue, and induce insulin resistance through increased insulin secretion. These metabolic effects may contribute to a higher risk of developing diabetes (17).

The significantly increased risk of hypertension in the TC with hypoP group highlights hypoparathyroidism as a potential risk factor for hypertension, possibly contributing to cardiovascular risk. However, no significant differences were observed in other cardiovascular outcomes, including stroke, ischemic heart disease, heart failure, or arrhythmia. Although hypoparathyroidism may influence blood pressure, its role in broader cardiovascular diseases warrants further investigation. In individuals with hypoparathyroidism, increased blood pressure and arterial stiffness have been observed, with a correlation between age and blood phosphorus levels (18). In addition, hypocalcemia can stimulate renin release, resulting in elevated angiotensin II and aldosterone levels, which promote sodium retention and vascular constriction (19).

Regarding renal outcomes, although the risk of urinary stones was significantly higher in the TC with hypoP group vs the control group, the risk of CKD did not differ significantly between the groups. Hypercalciuria, a known consequence of hypoparathyroidism, is a major risk factor for urinary stones (20). The increased risk of renal stones in women in the TC with hypoP group observed in this study suggests a potential sex-specific impact of hypoparathyroidism, which warrants further investigation.

The risk of cataracts was higher in the TC with hypoP group vs the control group, particularly in patients aged over 50 years. This is believed to result from the deposition of calcium phosphate salts on the lens (21). Long-term hypoparathyroidism and advanced age are risk factors for cataract progression (22).

In this study, we included 2 types of comparison groups: a general control group without TC or hypoparathyroidism, and a TC group without hypoparathyroidism following thyroid surgery to comprehensively analyze the risk of nonskeletal complications. However, thyroid stimulating hormone suppression therapy in patients with TC often leads to subclinical hyperthyroidism, which can independently affect the risk of nonskeletal complications. Additionally, the TC without hypoP group was relatively small compared with the control group, which may have limited statistical power of the comparisons with the TC with hypoP group. Nevertheless, these groups allowed us to partially address these limitations and present an in-depth assessment of the risk of nonskeletal complications in the TC with hypoP group, distinguishing this study from previous research.

This large cohort study systematically evaluated the risk of various nonskeletal complications in patients with TC and hypoparathyroidism. However, this study has some limitations. First, as this was a retrospective cohort study, establishing clear causal relationships was challenging. Second, certain confounding factors, including lifestyle, diet, physical activity, and medication use, may not have been fully controlled. While baseline physical activity data were available for a subset of participants, changes during follow-up could not be assessed, limiting the ability to evaluate its long-term effects on outcomes. Further prospective studies are recommended to confirm these associations and elucidate the underlying mechanisms. Third, owing to the reliance on the NHIS claims database, hypoparathyroidism was defined based on diagnostic codes and prescription durations rather than laboratory measurements, which may have limited the accuracy of hypoparathyroidism identification. Nevertheless, by defining hypoparathyroidism based on at least 3 repeated active vitamin D prescriptions over a period exceeding 180 days, we effectively excluded cases of transient postoperative hypoparathyroidism. In our cohort, 7.2% of patients with TC (430 out of 5928) were classified as having hypoparathyroidism based on this definition. Among them, 360 patients (6.1% of all patients with TC) continued active vitamin D for at least 12 months, aligning with the most recent management statement for postoperative permanent hypoparathyroidism (23). However, during the period our cohort was observed (2006-2019), a 6-month duration was widely used in clinical practice to distinguish chronic hypoparathyroidism from transient cases (24-26). While more recent guidelines suggest a 12-month threshold, this recommendation is an Un-GRADED statement based on expert consensus rather than high-quality evidence (23). As our study was designed using established clinical definitions at the time, our approach remains appropriate for evaluating long-term outcomes in this population. Additionally, over-the-counter vitamin D supplements, such as cholecalciferol, were not systematically recorded in the NHIS database. While this may have introduced some misclassification, prescription-based active vitamin D is the standard treatment for hypoparathyroidism in Korea, minimizing its potential impact on our findings. Fourth, the median follow-up period of our study was 5.07 years (IQR 2.19-7.38 years), which may be relatively short for fully assessing the long-term complications of hypoparathyroidism. Although similar large-scale studies have reported comparable follow-up durations, future longitudinal cohort studies with extended observation periods are warranted (27, 28). Finally, as this study was based on NHIS claims data, biochemical parameters including TSH and serum calcium levels, were not available, which may have limited the assessment of their contribution to cardiovascular outcomes.

In conclusion, the risk of cataracts was significantly higher in patients aged over 50 years, and urinary stones were more common among female patients in this group. These findings highlight the need for targeted monitoring and management strategies for nonskeletal complications in patients with TC and hypoparathyroidism, particularly in older adults and women. Additionally, hypertension was the only outcome that was significantly higher in the TC with hypoP group than in the TC without hypoP group, whereas the risks of diabetes mellitus and dyslipidemia were increased regardless of parathyroid function. This suggests that factors beyond hypoparathyroidism itself may contribute to the elevated risk of nonskeletal complications in patients with TC.

## Data Availability

The data that support the findings of this study are available from the National Health Insurance Service (NHIS) of Korea, but restrictions apply to the availability of these data, which were used under license for the current study and so are not publicly available. Data are, however, available from the authors upon reasonable request and with permission of the NHIS.

## Abbreviations

BMI: body mass index
CKD: chronic kidney disease
DBP: diastolic blood pressure
FPG: fasting plasma glucose
SBP: systolic blood pressure
TC: thyroid cancer
TC with hypoP: TC with hypoparathyroidism
TC without hypoP: TC without hypoparathyroidism

## References

[1] Kitahara CM, Sosa JA. The changing incidence of thyroid cancer. Nat Rev Endocrinol. 2016;12(11):646‐653. 27418023 10.1038/nrendo.2016.110 PMC10311569

[2] Abate EG, Clarke BL. Review of hypoparathyroidism. Front Endocrinol (Lausanne). 2016;7:172. 28138323 10.3389/fendo.2016.00172 PMC5237638

[3] Al-Azem H, Khan AA. Hypoparathyroidism. Best Pract Res Clin Endocrinol Metab. 2012;26(4):517‐522. 22863393 10.1016/j.beem.2012.01.004

[4] Clarke BL, Brown EM, Collins MT, et al Epidemiology and diagnosis of hypoparathyroidism. J Clin Endocrinol Metab. 2016;101(6):2284‐2299. 26943720 10.1210/jc.2015-3908 PMC5393595

[5] Underbjerg L, Sikjaer T, Mosekilde L, Rejnmark L. Cardiovascular and renal complications to postsurgical hypoparathyroidism: a danish nationwide controlled historic follow-up study. J Bone Miner Res. 2013;28(11):2277‐2285. 23661265 10.1002/jbmr.1979

[6] Underbjerg L, Sikjaer T, Mosekilde L, Rejnmark L. Postsurgical hypoparathyroidism–risk of fractures, psychiatric diseases, cancer, cataract, and infections. J Bone Miner Res. 2014;29(11):2504‐2510. 24806578 10.1002/jbmr.2273

[7] Swartling O, Evans M, Spelman T, et al Kidney complications and hospitalization in patients with chronic hypoparathyroidism: a cohort study in Sweden. J Clin Endocrinol Metab. 2022;107(10):e4098‐e4105. 35907259 10.1210/clinem/dgac456 PMC9516192

[8] Mazoni L, Matrone A, Apicella M, et al Renal complications and quality of life in postsurgical hypoparathyroidism: a case-control study. J Endocrinol Invest. 2022;45(3):573‐582. 34637114 10.1007/s40618-021-01686-2

[9] Ahn SH, Lee YJ, Hong S, et al Risk of fractures in thyroid cancer patients with postoperative hypoparathyroidism: a nationwide cohort study in Korea. J Bone Miner Res. 2023;38(9):1268‐1277. 37338940 10.1002/jbmr.4871

[10] Lee J, Lee JS, Park SH, Shin SA, Kim K. Cohort profile: the national health insurance service-national sample cohort (NHIS-NSC), South Korea. Int J Epidemiol. 2017;46(2):e15. 26822938 10.1093/ije/dyv319

[11] Cho SW, Kim JH, Choi HS, Ahn HY, Kim MK, Rhee EJ. Big data research in the field of endocrine diseases using the Korean National Health Information Database. Endocrinol Metab (Seoul). 2023;38(1):10‐24. 36758542 10.3803/EnM.2023.102 PMC10008661

[12] Ku EJ, Lee J, Yoo WS, Bae J, Lee EJ, Ahn HY. 2025. Long-term non-skeletal complications in patients with thyroid cancer with hypoparathyroidism following total thyroidectomy: a retrospective nationwide cohort study. Figshare. doi:10.6084/m9.figshare.27988343.v3. Published December 8, 2024.

[13] Austin PC . Balance diagnostics for comparing the distribution of baseline covariates between treatment groups in propensity-score matched samples. Stat Med. 2009;28(25):3083‐3107. 19757444 10.1002/sim.3697 PMC3472075

[14] VanderWeele TJ, Ding P. Sensitivity analysis in observational research: introducing the E-value. Ann Intern Med. 2017;167(4):268‐274. 28693043 10.7326/M16-2607

[15] Roh E, Noh E, Hwang SY, et al Increased risk of type 2 diabetes in patients with thyroid cancer after thyroidectomy: a nationwide cohort study. J Clin Endocrinol Metab. 2022;107(3):e1047‐e1056. 34718625 10.1210/clinem/dgab776

[16] Ahn HY, Lee J, Kang J, Lee EK. Increased risk of diabetes mellitus and hyperlipidemia in patients with differentiated thyroid cancer. Eur J Endocrinol. 2024;190(3):248‐255. 38536878 10.1093/ejendo/lvae026

[17] Eom YS, Wilson JR, Bernet VJ. Links between thyroid disorders and glucose homeostasis. Diabetes Metab J. 2022;46(2):239‐256. 35385635 10.4093/dmj.2022.0013 PMC8987680

[18] Pamuk N, Akkan T, Dağdeviren M, et al Central and peripheral blood pressures and arterial stiffness increase in hypoparathyroidism. Arch Endocrinol Metab. 2020;64(4):374‐382. 32267362 10.20945/2359-3997000000234 PMC10522079

[19] Villa-Etchegoyen C, Lombarte M, Matamoros N, Belizán JM, Cormick G. Mechanisms involved in the relationship between low calcium intake and high blood pressure. Nutrients. 2019;11(5):1112. 31109099 10.3390/nu11051112 PMC6566648

[20] Shoback DM, Bilezikian JP, Costa AG, et al Presentation of hypoparathyroidism: etiologies and clinical features. J Clin Endocrinol Metab. 2016;101(6):2300‐2312. 26943721 10.1210/jc.2015-3909

[21] Shoback D . Clinical practice. Hypoparathyroidism. N Engl J Med. 2008;359(4):391‐403. 18650515 10.1056/NEJMcp0803050

[22] Cherchir F, Oueslati I, Yazidi M, et al Long-term complications of permanent hypoparathyroidism in adults: prevalence and associated factors. Endocrine. 2024;84(3):1164‐1171. 38460072 10.1007/s12020-024-03765-9

[23] Khan AA, Bilezikian JP, Brandi ML, et al Evaluation and management of hypoparathyroidism summary statement and guidelines from the second international workshop. J Bone Miner Res. 2022;37(12):2568‐2585. 36054621 10.1002/jbmr.4691

[24] Bollerslev J, Rejnmark L, Marcocci C, et al European Society of Endocrinology Clinical Guideline: treatment of chronic hypoparathyroidism in adults. Eur J Endocrinol. 2015;173(2):G1-G 20. 26160136 10.1530/EJE-15-0628

[25] Brandi ML, Bilezikian JP, Shoback D, et al Management of hypoparathyroidism: summary statement and guidelines. J Clin Endocrinol Metab. 2016;101(6):2273‐2283. 26943719 10.1210/jc.2015-3907

[26] Orloff LA, Wiseman SM, Bernet VJ, et al American thyroid association statement on postoperative hypoparathyroidism: diagnosis, prevention, and management in adults. Thyroid. 2018;28(7):830‐841. 29848235 10.1089/thy.2017.0309

[27] Gosmanova EO, Chen K, Ketteler M, et al Risk of cardiovascular conditions in patients with chronic hypoparathyroidism: a retrospective cohort study. Adv Ther. 2021;38(8):4246‐4257. 34165700 10.1007/s12325-021-01787-7 PMC8342323

[28] Hamdy NAT, Decallonne B, Evenepoel P, Gruson D, van Vlokhoven-Verhaegh L. Burden of illness in patients with chronic hypoparathyroidism not adequately controlled with conventional therapy: a Belgium and The Netherlands survey. J Endocrinol Invest. 2021;44(7):1437‐1446. 33128157 10.1007/s40618-020-01442-y PMC8195792

